# Glutamate Afferents From the Medial Prefrontal Cortex Mediate Nucleus Accumbens Activation by Female Sexual Behavior

**DOI:** 10.3389/fnbeh.2019.00227

**Published:** 2019-10-04

**Authors:** Kelsey M. Moore, Wyatt L. Oelberg, M. Rose Glass, Matthew D. Johnson, Laura E. Been, Robert L. Meisel

**Affiliations:** ^1^Department of Neuroscience, University of Minnesota, Minneapolis, MN, United States; ^2^Department of Biomedical Engineering, College of Science and Engineering, University of Minnesota, Minneapolis, MN, United States; ^3^Department of Psychology, Haverford College, Haverford, PA, United States

**Keywords:** sexual behavior, reward, glutamate, medial prefrontal cortex, nucleus accumbens, DREADD

## Abstract

Low levels of desire and arousal are the primary sexual dysfunctions in women, necessitating neurobiological studies of sexual motivation in female animal models. As the mesocorticolimbic system is a primary neural circuit underlying sexual motivation, the goal of this study was to test the hypothesis that medial prefrontal cortex (mPFC) glutamate mediates sexual behavior activation of the nucleus accumbens. Glutamatergic neurons in the mPFC were activated by sex behavior, and these sex-activated cells shown to project to the nucleus accumbens. During sexual interactions with the male, glutamate transients recorded in the nucleus accumbens of female hamsters were specifically associated with the receipt of intromissions from the male. Further, inhibition of the mPFC during sex significantly decreased nucleus accumbens activation. Glutamatergic medial prefrontal cortical input to the nucleus accumbens mediates the activity in the nucleus accumbens during female sexual behavior. These results offer novel insights into the neurobiology of the motivational control of female sexual behavior and provide attractive avenues for pursuing target-specific and clinically-relevant therapies for sexual dysfunction in women.

## Introduction

Unlike in men where sexual dysfunction is primarily reflected in performance issues (i.e., erectile failure), sexual dysfunction in women is often more subtle, characterized by a loss of motivation to initiate sex and a loss of pleasure during sex (McCabe et al., 2016). This lack of interest in sex, especially among women in intimate relationships, carries with it serious psychological consequences that include relationship problems, low self-esteem, and importantly, decrements in health-related quality of life (Biddle et al., 2009). Unfortunately, there are few therapeutic options for women with low levels of sexual motivation, and available options are relatively ineffective (Jaspers et al., 2016; Goldstein et al., 2017). A reason so few therapeutic options exist comes from the erroneous belief that animal models cannot capture the essential components of sexual motivation in women. As a result, very little is known about neural mechanisms underlying female sexual desire in women.

In contrast to the view that female sexual desire cannot be modeled in animals, we and others have developed behavioral analyses that measure the incentive value and pleasurable consequences of female sexual behavior in rodents, including the female’s willingness to engage in copulatory activity with a male (Mendelson and Pfaus, 1989; Paredes and Vazquez, 1999; Meisel and Mullins, 2006; Cummings and Becker, 2012; Georgiadis et al., 2012). An important component of these behavioral analyses is the separation of the motivational components of female sexual behavior from the overt expression of female sexual behavior, i.e., the lordosis posture (Pfaus et al., 1990; Georgiadis et al., 2012). Hypothalamic circuits have long been known to mediate female sexual behavior in rodent models (e.g., Pfaff, 1980). More recent studies have pointed to the mesocorticolimbic circuits controlling female sexual motivation (Meisel and Mullins, 2006; Micevych and Meisel, 2017), consistent with the role of this system in motivational control in general (e.g., Salamone and Correa, 2012).

The nucleus accumbens has a key role in the incentive motivational processes (Bindra, 1969; Berridge, 2007) of both “wanting” and “liking” female sexual behavior (Micevych and Meisel, 2017). Damage to the nucleus accumbens of rats dramatically reduces the female’s willingness to engage in sexual interactions with a mounting male, i.e., wanting sex (Dohanich and McEwen, 1986; Rivas and Mir, 1991; Guarraci et al., 2002), though the lesions do not reduce the incidence of lordosis if the male is able to successfully mount the female. Separate studies link the nucleus accumbens to the rewarding consequences of sexual behavior for female rodents (Hedges et al., 2009; Been et al., 2013), indicating the role of this region in the liking of sexual behavior.

We have spent several decades studying sexual motivation in a female Syrian hamster model (reviewed in Meisel and Mullins, 2006). Syrian hamsters offer a distinct advantage over the more commonly used rat model. Early studies of the mesocorticolimbic circuits linked this system to locomotor activity (Mogenson et al., 1980). The primary limitation of using female rat sexual behavior to understand its control by this system is that female rats have a high level of locomotor activity during sexual interactions (e.g., Guarraci et al., 2002). This characteristic of female rat sexual behavior makes it very difficult to separate the locomotor activation of the mesocorticolimbic system from the activation resulting from sexual behavior. We explicitly chose female Syrian hamsters for our studies as they remain relatively immobile during sex, maintaining the lordosis posture for upwards of 9 min of a 10 min test (Meisel et al., 1988). As a result, it is much easier to associate mesocorticolimbic activation with components of sexual behavior in female Syrian hamsters than in female rats.

The neurobiological underpinnings of our model of the motivational control of female sexual behavior have focused on the mesocorticolimbic dopamine system and its innervation of the nucleus accumbens. We know that the pleasurable aspects of sexual behavior in female rodents and the willingness of these females to regulate sexual contacts with a male both correlate with nucleus accumbens dopamine release and are modulated by manipulations of dopamine innervation of the nucleus accumbens (Meisel and Mullins, 2006). At the same time, that dopamine is an important regulator of the nucleus accumbens with respect to female sexual behavior, concordant stimulation by both dopamine and glutamate is key to nucleus accumbens activity (Carlezon and Thomas, 2009). Research on male rats highlights the key role of mesocorticolimbic glutamate regulating copulation (Hernández-González et al., 2008; Beloate and Coolen, 2017), and in this light it is surprising that so little attention has been paid to the effects of glutamate in this system on female sexual behavior. In a similar vein, the medial prefrontal cortex (mPFC) and its glutamatergic inputs to the nucleus accumbens are at the core of this circuit, though very little is known about medial prefrontal cortical control of the nucleus accumbens during female sexual behavior.

In this report, we provide a converging set of experiments that identify for the first time the importance of mPFC glutamate innervation on the activation of the nucleus accumbens during female sexual behavior. We first analyzed c-Fos staining to evaluate neuronal activation in the mPFC and nucleus accumbens during sexual behavior in female Syrian hamsters. We next demonstrated that efferents from the mPFC to the nucleus accumbens core are activated during female sexual behavior. Further, c-Fos activation in the mPFC during sexual behavior was localized to glutamatergic neurons. *In vivo* recordings of extracellular glutamate in the nucleus accumbens were associated with the female’s receipt of intromission from the mounting male. Finally, we used viral expression of inhibitory DREADDs in the mPFC to demonstrate that silencing the mPFC during sexual behavior prevented the increase in nucleus accumbens c-Fos expression by female sexual behavior.

## Materials and Methods

### Animals

Adult (about 55 days old at arrival) female hamsters (Charles River Laboratories, Wilmington, MA, USA) were used as experimental subjects, whereas similar-aged adult male hamsters were used as stimulus animals for the sexual behavior tests. Females were housed individually and males housed in pairs in polycarbonate cages (females: 51 × 41 × 20 cm; males: 43 × 23 × 20 cm). The colony room was maintained on a reversed 14 h light/10 h dark photoperiod with lights off between 13:00 and 23:00. Behavioral testing was performed during the nocturnal animals’ dark phase. The animal room was maintained at 22°C, with food and water available for the animals *ad libitum* except during periods of behavioral testing. All procedures in these experiments were approved by the University of Minnesota IACUC and are in accordance with The Guide for the Care and Use of Laboratory Animals (NIH Publications No. 80-23; revised 2011).

### Surgeries

One week after arrival to the laboratory, female hamsters were bilaterally ovariectomized under sodium pentobarbital anesthesia (Nembutal, 8.5 mg/100 g body weight, i.p., Abbott Laboratories, Abbott Park, IL, USA). Stereotaxic surgery was performed directly following ovariectomy. Depending on the experiment, one of two stereotaxic approaches was taken. For the neural tracing study, unilateral intracranial injections were made by lowering a microinjection syringe (Model #701, Hamilton Company, Hamilton, Reno, NV, USA) under stereotaxic control (Microinjection Unit, Model 5002, David Kopf Instruments, Tujunga, CA, USA) into the NAc core and injecting a volume of 50 nL cholera toxin subunit β (CTB; Product #104, List Biological Laboratories, Campbell, CA, USA) over the course of 30 s. For viral vector delivery of an inhibitory DREADD, bilateral injections of 1.0 μL pAAV5-CaMKIIα-hM4D(Gi)-mCherry (Addgene, Cambridge, MA, USA) were infused over the course of 10 min. To minimize the flow of infused solution up the needle tract, the syringe was left in place for 10 min after each injection.

Female hamsters in the biosensor study were stereotaxically implanted with a unilateral BASi guide cannula (0.7 mm diameter; Bioanalaytical Systems, West Lafayette, IN, USA). The guide cannula was fixed to the skull using dental acrylic (Patterson Dental, St. Paul, MN, USA) extending to three stainless steel screws secured to the skull (Pinnacle Technology, Lawrence, KS, USA), and a stainless steel post was inserted into the cannula shaft to prevent occlusion.

Post-surgical analgesic (Butorphanol, 10 mg/kg, s.c., Fort Dodge Animal Health, Fort Dodge, IA, USA or meloxicam, 2 mg/kg, s.c., Norbrook, Overland Park, KS, USA) and antibiotic (0.1 mL Baytril, 2.27% solution s.c., Bayer Animal Health, Monheim, DE, USA) were provided on the day of surgery and for each of the next three postsurgical days for all animals.

### Sexual Behavior Testing

One or 3 weeks (viral vector studies) following surgery, female hamsters were hormone-primed for sexual behavior testing *via* subcutaneous injections of 10 μg of estradiol benzoate (Sigma-Aldrich, St. Louis, MO, USA) in 0.1 mL of cottonseed oil (Sigma-Aldrich) at approximately 48 and 24 h prior to the sexual behavior test, followed by a subcutaneous injection of progesterone (500 μg in 0.1 mL of cottonseed oil, Sigma-Aldrich) 4 h prior to the testing. Females were paired with a male hamster in either the biosensor testing chamber or in the female’s home cage for a 10 min session. Copulatory parameters of the females (lordosis latency and total lordosis duration) and males (mounts, intromissions, ejaculations) were obtained to ensure that the females received comparable levels of sexual stimuli. For c-Fos experiments, control females were not given a sexual behavior test following hormonal priming; instead their cage was placed in the same behavioral testing room with the male hamsters present for 10 min. In the DREADD experiment, female hamsters were given either 5 mg/kg CNO in 0.9% saline (Enzo Life Sciences, Farmingdale, NY, USA) or an equivalent volume of saline (0.1 mL/100 g body weight) 30 min prior to behavioral testing.

### Perfusion and Tissue Sectioning

Sixty minutes after sexual behavior testing or soon after biosensor recordings, female hamsters were deeply anesthetized with the euthanizing agent Buthanasia-D (0.2 mL i.p., Merck Animal Health, Summit, NJ, USA) and transcardially perfused with 25 mM phosphate buffer (~50 mL) containing 0.9% saline (PBS, pH = 7.6) followed by 4% paraformaldehyde in PBS (~500 mL). The brains were removed and post-fixed for either 2 h or overnight (c-Fos/CTB experiment only) in 4% paraformaldehyde and then placed in a 10% sucrose solution in PBS overnight. Serial coronal sections (40 μm) of frozen brain tissue were sectioned on a microtome (American Optical, model 860) and every fourth section was processed for immunohistochemical localization of the proteins of interest.

### Immunohistochemistry

#### c-Fos Staining

Free-floating sections were rinsed in PBS with 0.1% IgG-free bovine serum albumin (Jackson ImmunoResearch, West Grove, PA, USA) wash buffer and then incubated in a polyclonal antibody to c-Fos primary (1:10,000, Santa Cruz Biotechnology, Santa Cruz, CA, USA, Cat# sc-52) in wash buffer with 0.3% Triton-X100 (Sigma-Aldrich) at room temperature for 48 h at 4°C. After rinsing in wash buffer, sections were incubated for 60 min at room temperature in biotinylated anti-rabbit IgG secondary antibody (1:600; Vectastain Elite ABC kit; Vector Laboratories, Burlingame, CA, USA), rinsed in wash buffer, and then incubated in an avidin-biotin horseradish peroxidase complex (1:200, Vectastain Elite ABC Kit) for 60 min at room temperature. The sections were then rinsed in wash buffer and reacted in a 0.1 M Tris buffer (pH = 7.6) with 0.56 mM 3,3′-diaminobenzidine tetrahydrochloride (DAB, Sigma-Aldrich) solution, 0.003% hydrogen peroxide and 63 mM nickel ammonium sulfate (Sigma-Aldrich). After 5 min, sections were rinsed in Tris buffer to stop the chromagen reaction. Immunostained sections were mounted onto glass slides (Adhesion Superfrost Plus Microscope Slides, Brain Research Laboratories, Newton, MA, USA), cover slipped with DPX mounting media (Sigma-Aldrich), and imaged using bright field microscopy.

#### Dual Labeling for c-Fos With GAD or CaMKIIα

Free-floating sections were treated as described for c-Fos staining. After the DAB reaction and appropriate rinses, sections were incubated for 72 h at 4°C in a primary antibody against GAD67 (1:3,000, EMD Millipore, Burlington, MA, USA, Cat#MAB5406) or for 48 h at 4°C in a primary antibody against CaMKIIα (1:1,000, Abcam, Cambridge, UK, Cat# ab111890), and rinsed as described. The sections were then incubated in secondary antibody for 1 h followed by avidin-biotinylated HRP complex for 1 h with appropriate rinses. Finally, sections were incubated a second time in DAB, though without the nickel ammonium sulfate, washed, mounted on slides, and coverslipped.

#### Dual Labeling for c-Fos With CTB

Free-floating sections were treated as described for c-Fos staining. After the DAB reaction and appropriate rinses, sections were incubated for 48 h at 4°C in a primary antibody against CTB (1:70,000, List Biological Laboratories, Campbell, CA, USA, Cat# 703) and rinsed. The sections were then incubated in secondary antibody for 1 h followed by avidin-biotinylated HRP complex for 1 h with appropriate rinses. Then sections were incubated for 5 min in DAB in 0.175 M sodium acetate buffer without nickel ammonium sulfate, washed, mounted on slides, and coverslipped.

### Viral Vector Visualization and Fluorescent Histochemistry

Sixty minutes following the sexual experience, subjects were injected with an overdose of Buthanasia-D, intracardially perfused and the brains sectioned as described in “Perfusion and Tissue Sectioning” section. Free-floating sections were rinsed in PBS wash buffer and then incubated in anti-c-Fos (1:10,000) in wash buffer with 0.3% Triton-X100 (Sigma-Aldrich) at 4°C for 24 h. After rinsing in wash buffer, sections were incubated in a biotinylated secondary antibody (1:600, Vector Laboratories) for 60 min at room temperature, rinsed in wash buffer, and then incubated in streptavidin DyLight^®^ 488 (1:200, SA-5488, Vector Laboratories) for 60 min at room temperature. Sections were then rinsed in wash buffer before being mounted onto glass slides, cover slipped with VectaShield^®^ HardSet™ mounting medium (H-1500, Vector Laboratories) and examined under a confocal microscope (Leica SPE personal confocal, Wetzlar, Germany) for c-Fos localization as well as the rostral-caudal spread of AAV injection, visualized directly with the AAV-expressed mCherry.

### Image Analysis

Brightfield microscopic analyses of regions of interest in the mPFC and the NAc were obtained with a Leica microscope (Leica DN4000 B) equipped with a digital camera connected to a computer running Leica software. Our approach to counting fields of stained neurons was modeled after Bradley and Meisel (2001). Digital images were obtained and adjusted to match brightness and contrast as seen through the microscope. The same digital settings were used to capture all images for an individual experiment. The digital images were then opened in Adobe Photoshop (Adobe, San Jose, CA, USA) to place boxes identifying the regions of interest to analyze.

Analyses for the caudal NAc were based on Bradley and Meisel’s (2001) findings. Within rostral to caudal coronal sections containing the NAc, the anterior commissure is monotonically shifted in a medial direction. We can take advantage of this to precisely identify a rostral-caudal level to analyze that is matched across all brains. For the NAc, we took a single section in which the distance from the ventral tip of the lateral ventricle was 300 μm from the medial edge of the anterior commissure, and from this section took cell counts from the right hemisphere in each animal. For the mPFC a single section was also measured to be consistent with the approach for the NAc. In this case, the section to be counted represented the mid rostral-caudal level of the mPFC and was matched to a template histological section based on the position and shape of the corpus collosum to ensure that the region of interest was sampled from the same location among all brains analyzed. A rectangular box was placed within the region of interest to ensure a consistent area in which cell counts were obtained. ImageJ Fiji software (Schindelin et al., 2012) was used to count labeled cells within these regions.

Fluorescent images were obtained with a Leica SPE personal confocal microscope for c-Fos localization as well as for parameters of the AAV injection. For all cell counts, digital images of the regions of interest were transferred to Photoshop to superimpose the rectangle outlining the counting region and the labeled cells were counted manually using the ImageJ cell-counter plugin.

### Biosensor Testing

Glutamate oxidase-based sensors (Pinnacle Technology, Lawrence, KS, USA) were used to detect glutamate release in the female hamsters during sexual behavior (Moore et al., 2017). First, sensors were calibrated before use. Animals were then lightly anesthetized using a subthreshold dose of sodium pentobarbital (Nembutal, 3 mg/100 g body weight, i.p.) and the calibrated sensor was inserted through the guide cannula. Each animal was then placed in the testing chamber consisting of a 10-gallon glass aquarium with pine bedding taken from the animal’s home cage, after which the sensor was connected to a potentiostat *via* an electrically shielded cable and electrical swivel (Pinnacle Technology). After the animal was injected with progesterone, the sensor was allowed to equilibrate for the next 4 h. A male hamster was then placed in the testing chamber for approximately 10 min while the amperometric signal and time-locked video data were simultaneously recorded. During sexual interactions with a male, female hamsters maintain a tonic lordosis posture for minutes on end while the male intermittently mounts the female (e.g., Meisel et al., 1988). Consequently, we were able to measure glutamate transients in the female’s brain in response to discrete mating stimulation provided by the male hamster and in the absence of non-specific movement artifacts.

After recordings were completed, females were perfused as described, the brains frozen sectioned at 40 μm, and the free floating sections stained conventionally with cresyl violet acetate. The stained sections were mounted on slides and coverslipped. Brightfield images of the location of the biosensor were obtained with a Leica microscope (Leica DN4000 B) and the digital images used to plot the location of the biosensor.

### Biosensor Analyses

#### Finding Peaks

After importing the raw amperometric data and behavioral annotations acquired during experimental recording, the raw current vs. time was plotted. A moving average taken over 20 data points (one point collected each second) was then used to create a smoothed data set from the raw voltage; this represented more tonic changes occurring throughout the experimental recording. Then, to obtain a normalized signal producing a flat basal response, the smoothed dataset was subtracted from the raw dataset. Using the standard “findpeaks” MATLAB function, we located peaks in the data using a threshold value determined by calculating half of the root-mean-square (RMS) of the normalized signal.

#### Peak Characterization

Peak characterization was performed by examining both the peak prominence in units of amplitude (nanoamps) and the width of the peak in units of time (seconds). Both the average peak prominence and width were compared across each subsequent mating bout using a repeated measures analysis of variance (ANOVA) to determine if tonic changes occurred during the course of experimental testing. A peri-peak analysis was also performed using combined data from all mating bouts using frequency histograms of the number of peaks that occurred within a 5 s window of each behavioral annotation (e.g., mount or mount with subsequent intromission). Peri-event time analyses between mounts with and without intromission were also performed that included the first 5 min after the start of the first mating bout.

#### Statistics

Parametric statistical tests were based on the demonstration of homogeneity of variance among treatment groups. For the immunohistological experiments, unpaired *t*-tests were used to evaluate possible differences between animals receiving sexual behavior testing and untested controls. Proportions of mounts with and without intromission associated with glutamate peaks for individual animals were compared by Chi-squared tests using the online Graph Pad 2 × 2 contingency table calculator^1^. One-way ANOVAs were used to analyze the DREADD experiment, with Tukey multiple comparison *post hoc* tests probing significant ANOVAs. All significant differences were based on *p* < 0.05.

## Results

### Female Sexual Behavior Activates the NAc and mPFC

**Figure 1:**
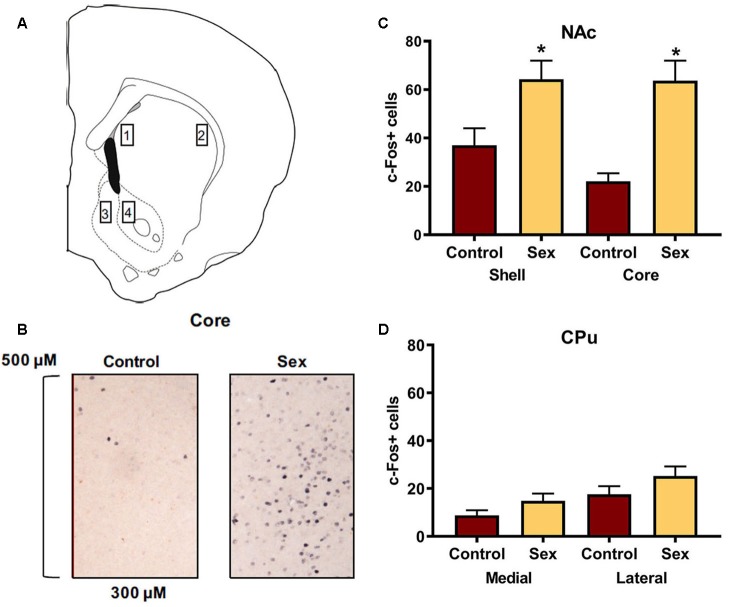
Female sexual behavior activates the nucleus accumbens (NAc) shell and core, but not the medial or lateral caudate-putamen (CPu). **(A)** Hamster atlas plate (+2.1 mm from Bregma) indicating counting domains for 1-medial CPu, 2-lateral CPu, 3-NAc shell, and 4-NAc core. **(B)** Representative c-Fos counting boxes for the NAc core. **(C)** There was no significant difference between animals that received sex experience and controls in either the medial or lateral CPu. **(D)** There were significantly more c-Fos positive cells in animals that received sex experience than for their control counterparts in both the shell and core of the NAc (*indicates *p* < 0.05).

Figure 1A identifies the locations for the regions of interest in which c-Fos cells were counted in the dorsal and ventral striatum. An example of c-Fos staining is illustrated in Figure 1B. Confirming previous findings, female hamsters (*n* = 14) who received a 10 min sexual behavior test had significantly more c-Fos positive cells in the NAc core compared with control female hamsters (*n* = 12) who remained in their cage in the presence of male hamsters (*t*_(24)_ = 4.41, *p* < 0.0002, Figure 1D). A similar increase in c-Fos labeling was observed in the NAc shell of these animals (*t*_(24)_ = 2.59, *p* < 0.02, Figure 1D). We used the caudate as an anatomical control and did not find any change in c-Fos labeling in the medial or lateral caudate as a function of sex behavior testing (Figure 1C).

**Figure 2:**
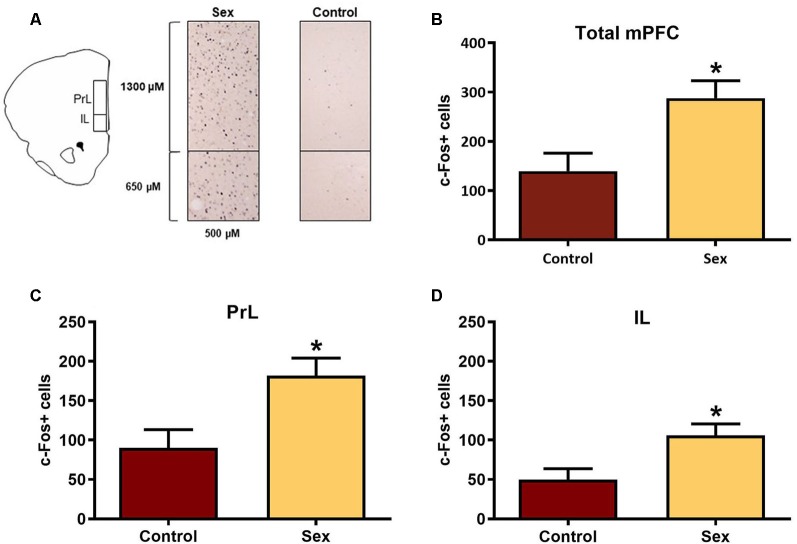
Female sexual behavior activates the medial prefrontal cortex (mPFC). **(A)** Hamster atlas plate (+3.2 mm from Bregma) counting domains for the prelimbic (PrL) and infralimbic (IL) regions of the mPFC. **(B)** There was a significant increase in c-Fos labeled cells after sex behavior in the combined regions of the mPFC (Total mPFC), which was represented individually in both the **(C)** PrL and **(D)** IL subregions (*p < 0.01).

The mPFC is part of the mesocorticolimbic circuit that includes the nucleus accumbens, yet this region had not been examined previously with respect to activation by female sexual behavior. Figure 2A illustrates the regions of interest for the c-Fos analyses in the mPFC. Females receiving a sex behavior test (*n* = 15) had significantly more total c-Fos labeled cells (*t*_(26)_ = 2.90, *p* < 0.01, Figure 2B) and more c-Fos labeled cells in both the infralimbic (*t*_(26)_ = 2.73, *p* < 0.01, Figure 2C) and prelimbic (*t*_(26)_ = 2.86, *p* < 0.01, Figures 2B,D) subdivisions of the mPFC when compared with female hamsters who remained in their cage in the presence of male hamsters (*n* = 13).

### Sex-Activated mPFC Neurons Are Glutamatergic

Given our finding of activation of the mPFC following female sexual behavior, we wished to determine the neuronal phenotype of these sex-activated cells. We used double label immunohistochemistry to identify sex-activated GABAergic and glutamatergic cells in this region. After confirming equal numbers of GAD labeled neurons in female hamsters tested for sexual behavior (*n* = 6) or control females (*n* = 7), we found no difference in the number of c-Fos positive cells between these groups (data not shown).

**Figure 3:**
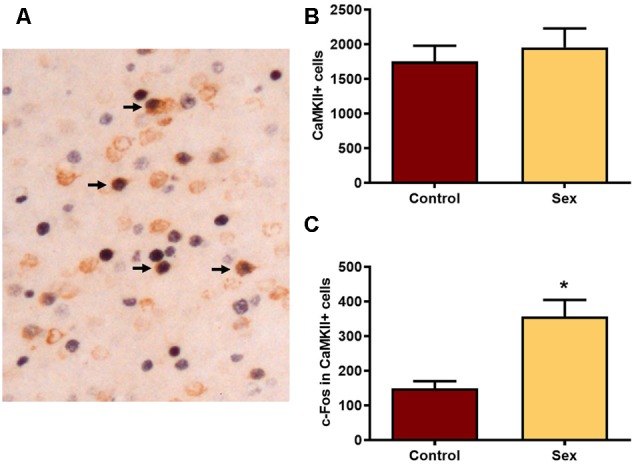
Female sexual behavior increases the number of c-Fos neurons in CaMKIIα neurons in the mPFC. **(A)** A histological image illustrating the double labeling for c-Fos and CaMKIIα. Arrows point to several double labeled cells in which nuclear staining for c-Fos is a black reaction product and cytoplasmic CaMKIIα is brown. **(B)** There were no differences in the total numbers of CaMKIIα labeled neurons in the total mPFC following sex behavior. **(C)** Sex behavior increased the number of c-Fos cells within CaMKIIα neurons for the total mPFC****, demonstrating increased activation of PFC glutamatergic neurons following a single sexual experience (*indicates *p* < 0.01).

We next used CaMKIIα labeled cells (Figure 3A) in the mPFC as a marker for glutamatergic neurons. There were no group differences in the numbers of CaMKIIα labeled cells (Figure 3B) between females receiving sexual behavior testing (*n* = 6) or control females (*n* = 7). Across all levels of the mPFC female sexual behavior increased the number of c-Fos labeled CaMKIIα positive cells compared to controls (*t*_(11)_ = 4.20, *p* < 0.002; Figure 3C). These results indicate that female sexual behavior activates mPFC glutamatergic neurons.

### Characterization of Sex-Activated Afferents to the NAc Core

**Figure 4:**
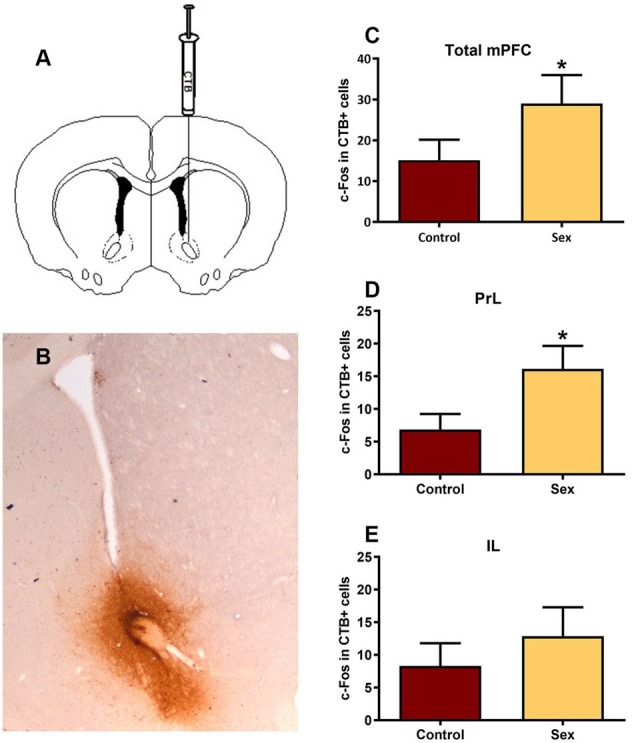
Double immunolabeling revealed a significant increase in activated inputs to the NAc core from the prelimbic region of the mPFC. **(A)** Illustration of the unilateral infusion of cholera toxin B (CTB) targeted to the core of the NAc. **(B)** Immunohistochemical labeling of the retrograde tracer CTB confirmed injection sites were biased to the core of the NAc. **(C)** There was no significant change in c-Fos labeling in CTB cells within the total mPFC, though **(D)** there was a significant increase in c-Fos labeling within CTB cells in the prelimbic (PrL) subregion following sex behavior. **(E)** The infralimbic (IL) region of the mPFC did not exhibit a change in c-Fos within CTB labeled cells following sex (*p < 0.05).

After identifying the neurotransmitter phenotype of these sexual behavior-activated mPFC neurons, we directly mapped the underlying circuitry using dual labeling of the retrograde tracer cholera toxin B (CTB, Figure 4) and c-Fos to determine active afferents to the NAc during sex. Immunohistochemical analysis revealed that all of the CTB tracer injections were core biased in the NAc (Figure 4A). mPFC neurons providing afferents to the NAc core had increased numbers of c-Fos labeled cells (Figure 4B) in females tested for sexual behavior (*n* = 15) compared with control females (*n* = 13), with an increase in the prelimbic (*t*_(26)_ = 2.11, *p* < 0.05, Figure 4C), but not infralimbic (Figure 4D) portions of the mPFC.

### Characterization of NAc Glutamate Release During Sexual Behavior

Given that sexual behavior activated glutamatergic neurons in the mPFC that project to the NAc, we used glutamate biosensor recordings to test whether there were elevations in glutamate in the female hamster NAc during sexual interactions with a male. Our hypothesis was that glutamate would be elevated during sex, with the specific expectation that there would be glutamate transients associated with the receipt of copulatory stimulation from the mounting male hamster. Hence for this experiment, the focus of our analyses was on individual biosensor cases rather than on the grouped data.

#### NAc Core

**Figure 5:**
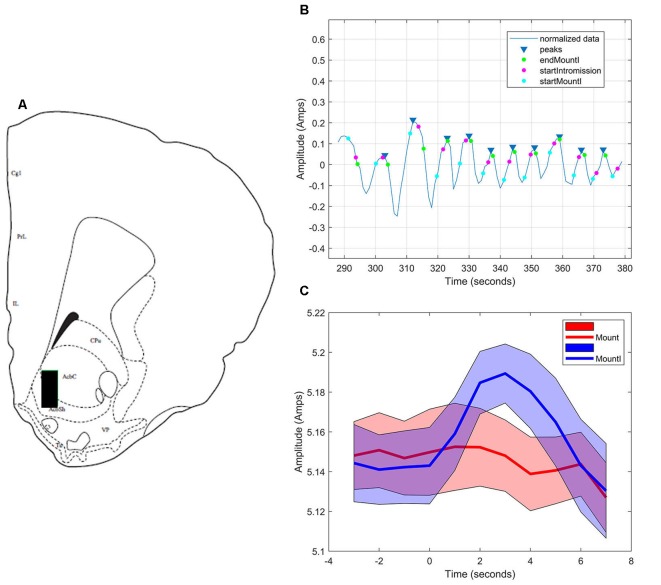
Glutamate biosensor recording in NAc core, Subject #1.** (A)** This probe was in the center of the rostral NAc core (+2.6 mm from Bregma). **(B)** MATLAB annotated signal and peak analysis for the first mating bout. Blue rectangles indicate peaks determined by a threshold value of half of the root-mean-square (RMS) of the normalized signal. Blue circles (startMountI) indicate the start of a mount that results in an intromission. Magenta circles (startIntromission) indicate the start of a penile intromission from a male. Green circles (endMountI) indicate the end of a mount that resulted in an intromission. **(C)** Mounts with subsequent intromissions were collapsed across the first 5 min of the sex test. These mounts with intromission had a coincident increase in glutamate (blue signal) that peaked 3 s after the start of the mount. No coincident signal was seen in glutamate among mounts without intromission (red signal).

During sexual interactions with the male hamster, glutamate transients were regularly recorded in NAc core biosensor placements (*n* = 4). Figure 5A demonstrates one representative case (see Supplementary Figures S1–S3 for additional core cases). The glutamate transients were differentially associated with the receipt of intromission by the mounting male hamster (Figure 5B). In this female hamster (and in two of the three cases reported in the Supplemental Results) there were significantly more peaks within 5 s of the start of a mount that resulted in intromission than for mounts without a subsequent intromission (see Table 1). The temporal patterning of these peaks was consistent among all animals tested, as illustrated in Figure 5C with all peaks occurring within 3 s of the start of a mount with intromission. Very few glutamate peaks were measured in conjunction with mounts without subsequent intromission, and for these peaks there was no concordance in the timing of the fluctuations in the glutamate signal (Figure 5C).

**Table 1:** Proportion of mounts with and without subsequent intromission associated within 5 s of a glutamate peak in NAc core.

Subject	Mounts w/Intromission	Mounts alone	Chi Square analysis
NAcC-1	49/53 (93%)	16/52 (31%)	χ^2^ = 42.3, *p* < 0.001
NAcC-2	29/45 (64%)	0/5 (0%)	χ^2^ = 7.7, *p* < 0.01
NAcC-3	23/27 (85%)	12/21 (57%)	χ^2^ = 4.7, *p* < 0.05
NAcC-4	10/14 (71%)	1/4 (25%)	χ^2^ = 2.8, NS

#### NAc Shell

**Figure 6:**
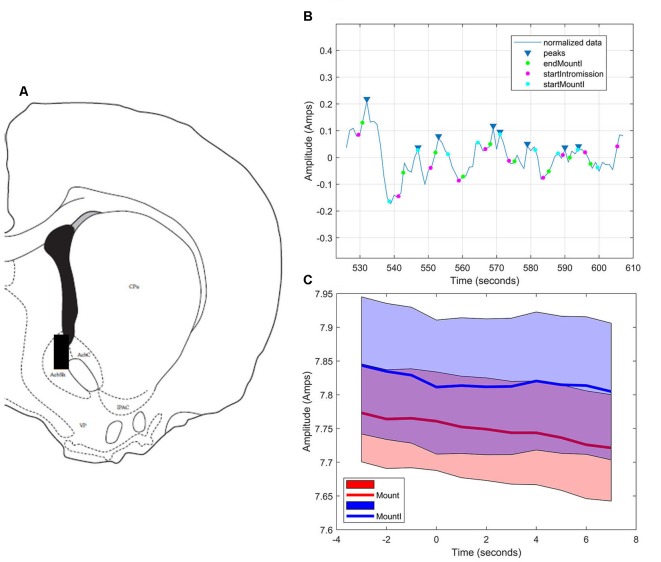
Glutamate biosensor recording in NAc shell, Subject #1.** (A)** This probe was in the caudal NAc shell (+1.5 mm from Bregma). **(B)** MATLAB annotated signal and peak analysis for the first mating bout. Blue rectangles indicate peaks determined by a threshold value of half of the RMS of the normalized signal. Blue circles (startMountI) indicate the start of a mount that results in an intromission. Magenta circles (startIntromission) indicate the start of a penile intromission from a male. Green circles (endMountI) indicate the end of a mount that resulted in an intromission. **(C)** Although there are significantly more glutamate peaks associated with mounts that resulted in intromission, there was no coincident timing of those peaks (blue signal), a finding similar to that of mounts without intromission (red signal).

As in the NAc core, glutamate transients were found in NAc shell recordings (*n* = 4). Figures 6A,B demonstrates one representative case (see Supplementary Figures S4–S6 for additional shell cases 2–4). In each case, there were significantly more peaks within 5 s of the start of a mount that resulted in intromission than for mounts without a subsequent intromission (see Table 2). In contrast to glutamate activity in the NAc core during sexual interactions, in the shell there was no concordance in the timing of the glutamate peaks relative the start of mounts with intromission (Figure 6C).

**Table 2:** Proportion of mounts with and without subsequent intromission associated within 5 s of a glutamate peak in the NAc shell.

Subject	Mounts w/Intromission	Mounts alone	Chi Square analysis
NAcSh-1	52/58 (90%)	5/31(16%)	χ^2^ = 47.4, *p* < 0.001
NAcSh-2	38/40 (95%)	3/48 (6%)	χ^2^ = 69.1, *p* < 0.001
NAcSh-3	35/41(85%)	6/45 (13%)	χ^2^ = 44.6, *p* < 0.001
NAcSh-4	45/45 (100%)	6/30 (20%)	χ^2^ = 52.9, *p* < 0.001

#### Medial Caudate

**Figure 7:**
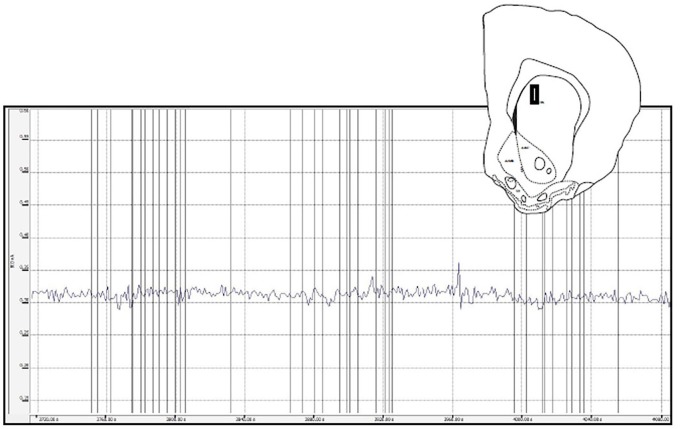
This figure depicts a glutamate biosensor placement within the medial caudate (insert) as well as the raw trace for the glutamate signal during a sex behavior test. For this and the other caudate animals there were few peaks recorded and no systematic glutamate changes associated with behavioral events (vertical lines).

There were no peaks identified in any of the recordings in association with behavioral events for any of the four hamsters with probes in the medial caudate used as anatomical controls (Figure 7).

### Inhibitory DREADD Silencing of mPFC During Sexual Behavior

**Figure 8:**
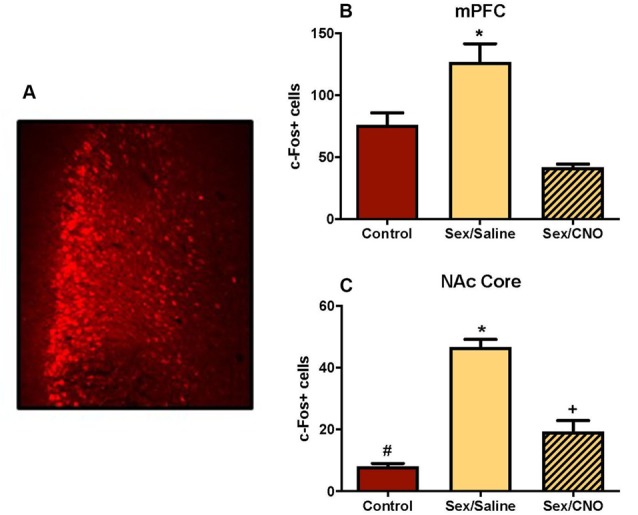
Silencing glutamatergic mPFC projections decreases NAc c-Fos in response to sex. **(A)** An example of a mPFC injection sites identified based on the location of the mCherry reporter. **(B)** Female sexual behavior significantly increased c-Fos stained cells within the mPFC, an effect that was blocked by pretreatment with CNO (*indicates *p* < 0.05).** (C)** Female sexual behavior increased c-Fos labeling in the core of the NAc, an effect that was similarly blocked by DREADD inhibition of the mPFC. Each group is significantly different from each other as indicated by distinct symbols (*p* < 0.05).

In this study we used viral expression of DREADDs to silence glutamatergic mPFC neurons in female hamsters during sexual behavior to test whether c-Fos activation in the NAc core was driven by mPFC glutamatergic afferents. Because the sex-activated neurons in the mPFC were CaMKIIα-expressing neurons (Figure 3D), we inhibited the mPFC using an inhibitory DREADD (Roth, 2016) with a CaMKIIα promoter. Viral injection sites were verified as being localized to the mPFC using fluorescent microscopy for the mCherry reporter. Only AAV-injections that spanned the prelimbic and infralimbic subdivisions of the mPFC were included in the analyses (Figure 8A). Control females not receiving sexual behavior received either a saline injection or CNO. There were no differences between saline and CNO animals in this condition (data not shown), so these sex behavior controls (*n* = 6) were combined into a single treatment group. Analyses of the mPFC confirmed that the CNO decreased c-Fos labeling following sexual behavior. There was a significant treatment effect across groups (*F*_(2,15)_ = 16.64, *p* < 0.001; Figure 8B), with *post hoc* Tukey’s multiple comparisons tests confirming that saline-treated females tested for sexual behavior (*n* = 6) had more c-Fos labeling in the mPFC than did control females not tested for sexual behavior (*p* < 0.01). Females tested for sexual behavior and receiving CNO (*n* = 6) had fewer c-Fos labeled cells than did the saline-treated females receiving sex (*p* < 0.01). There was no significant difference between the no sex controls and sex tested females receiving CNO.

Key to our overarching hypothesis, this inhibition of the mPFC resulted in significantly attenuated c-Fos labeling in the NAc core following female sexual behavior (*F*_(2,15)_ = 60.76, *p* < 0.0001; Figure 8C) with *post hoc* Tukey’s multiple comparisons tests confirming that saline-treated females tested for sexual behavior had more c-Fos labeling in the NAc core than did control females not tested for sexual behavior (*p* < 0.01). Females tested for sexual behavior and receiving CNO had fewer c-Fos labeled cells than did the saline-treated females receiving sex (*p* < 0.01), but were not reduced to control levels, with these groups demonstrating a significant difference from each other (*p* < 0.01). The CNO injections did not affect the levels of lordosis shown by the hamsters (data not shown), so the effects of the CNO on c-Fos in the NAc were independent of the levels of sexual behavior displayed by the female hamsters. These results suggest that glutamate neurons in the medial PFC activated during sex are at least partially responsible for driving neuronal excitability in the NAc.

## Discussion

Despite a large literature detailing dopaminergic neurotransmission in mesolimbic circuitry during female sexual behavior (Meisel et al., 1993; Mitchell and Gratton, 1994; Mermelstein and Becker, 1995; Kohlert et al., 1997; Kohlert and Meisel, 1999; Becker et al., 2001; Jenkins and Becker, 2003), the role of glutamate has been disproportionately understudied. The overarching goal of this study was to help close this gap in knowledge, adding to our understanding of the complex underpinnings of female sexual reward and motivation.

Our discovery that the mPFC (see Wise, 2008 for a discussion of the prefrontal cortex in rodents) is activated by sexual behavior led us to consider the functional relationship between the mPFC and the nucleus accumbens with respect to the control of female sexual behavior. The ventral tegmental area (VTA) sends distinct dopaminergic projections to both the mPFC and the NAc, rather than collateral inputs to each region (Swanson, 1982; Lammel et al., 2008; Yetnikoff et al., 2014). Based on this pattern of innervation, we explored the serial connection between the mPFC and NAc in conjunction with activation by female sexual behavior. Our results demonstrate that sexual behavior had no effect on the numbers of glutamate neurons in the mPFC (as measured by CaMKIIα staining). Instead, the existing glutamate neurons within the mPFC were activated by female sexual behavior, and these activated neurons project to the NAc. Finally, DREADD-mediated inhibition of glutamatergic mPFC neurons prevented increased activity in the NAc following sex, providing converging evidence of the importance of mPFC glutamatergic projections driving activity in the NAc. What is missing from this work, and is a focus of ongoing research, is what behavioral functions are affected by inhibition of PFC glutamatergic inputs to the NAc. We found that the expression of lordosis is not affected by direct inhibition of the PFC (and in turn indirect inhibition of the NAc). Our next step is to test different motivational endpoints to see whether these behaviors are mediated by PFC glutamatergic activation of the NAc.

In addition to anatomically demonstrating the involvement of prefrontal glutamatergic neurotransmission in female sexual behavior, we sought to characterize glutamate release in the NAc during sexual behavior. Using enzymatic biosensors, we discovered that the core of the NAc releases glutamate with a short latency preceding individual intromissions from the male. These glutamate peaks had a coincident timing with respect to the onset of intromission. These results are consistent with the view that one function of the nucleus accumbens core is to coordinate sensory cues (in this case intromission by the mounting male) with an appropriate action, a process not affected by repeated presentation of the stimulus (e.g., Brown et al., 2011). Intromission also elicited glutamate transients in the NAc shell, though these peaks were not coordinated in time. We hypothesize that the shell is responding to the rewarding consequences of intromission, a process that is more abstract and altered by repeated exposure to the stimulus, and therefore is more variable in its timing (e.g., Brown et al., 2011; Sackett et al., 2017).

The timing of the onset of glutamate release was an interesting finding that came from the characterization of glutamate release in the NAc during sexual behavior. Although the peak of the transient occurred after the onset of a mount culminating in intromission, the initial rise in glutamate was found to actually precede the actual receipt of intromission. We and others have demonstrated that female hamsters can control whether the mounting male hamster can achieve intromission (Noble, 1979, 1980; Bradley et al., 2005; Parada et al., 2014). These data suggest that glutamate in the nucleus accumbens is signaling this anticipatory response. A higher proportion of glutamate peaks occur in response to mounts with subsequent intromission as opposed to mounts alone, though a smaller proportion of mounts that do not result in intromission are also associated with glutamate transients. This small proportion of mounts without subsequent intromission that are still associated with glutamate release may indicate prediction error by the female (Hart et al., 2014; Saddoris et al., 2015; Gmaz et al., 2018).

In these experiments, we focused on the role of glutamate innervation mediating the effects of sex-induced NAc activation; however, we know that the NAc receives both dopamine and glutamate functional inputs (Zahm and Brog, 1992; Brog et al., 1993; O’Donnell and Grace, 1995; Kelley, 2004; Britt et al., 2012). Dopamine functions as a neuromodulator within the NAc coordinating with glutamatergic afferents to regulate the excitability of NAc neurons (O’Donnell et al., 1999; Nicola et al., 2000). Dopamine can affect intracellular signaling to modulate the functional responsiveness of both metabotropic and ionotropic glutamate receptors (Chen and Roche, 2007; Cahill et al., 2014). Understanding the coordinated actions of both dopamine and glutamate on nucleus accumbens neurons in regulating female sexual behavior is a goal of our current research.

Consistent with the literature from rodent models, mesocorticolimbic circuitry mediates the rewarding consequences of sexual behavior in people (Stahl, 2010; Georgiadis and Kringelbach, 2012; Oei et al., 2012; Kingsberg et al., 2015). Increased PFC and nucleus accumbens activation has been demonstrated in response to different components of sexual arousal in human subjects (Bocher et al., 2001; Sabatinelli et al., 2007; Voon et al., 2014; Wehrum-Osinsky et al., 2014; Lee et al., 2015; Ruesink and Georgiadis, 2017). Within this circuitry, dopamine and glutamate are the predominant signaling molecules, though how dopamine or glutamate release may be associated with sexual function or dysfunction in women is unknown. It is abundantly clear that animal models of sexual reward and motivation provide a logical preclinical avenue to a mechanistic understanding for the development of targeted therapeutics (Kingsberg et al., 2015; Jaspers et al., 2016), despite obvious differences in behavior patterns between animals and people. In this context, we have developed behavioral tests to measure both the pleasurable consequences of sexual interactions in female hamsters as well as the motivational control (Meisel and Mullins, 2006). The emphasis for future studies will be to evaluate neuronal mechanisms underlying sexual motivation and pleasure in our Syrian hamster model to provide viable directions for the development of therapeutics for low sexual desire in women.

## Data Availability Statement

The datasets generated for this study are available on request to the corresponding author.

## Ethics Statement

The animal study was reviewed and approved by University of Minnesota Institutional Animal Care and Use Committee.

## Author Contributions

LB, RM and KM: project conception and experimental design. LB, MG and KM: execution of experiments. LB and KM: data analyses. MJ and WO: Matlab analyses of biosensor data. LB, RM, KM and WO: manuscript preparation.

## Conflict of Interest

The authors declare that the research was conducted in the absence of any commercial or financial relationships that could be construed as a potential conflict of interest.

## Footnotes

^1^https://www.graphpad.com/quickcalcs/contingency2/
