# Supplementary material for: Glutamate Afferents From the Medial Prefrontal Cortex Mediate Nucleus Accumbens Activation by Female Sexual Behavior
Source: Front Behav Neurosci. 2019 Oct 4;13:227. doi: 10.3389/fnbeh.2019.00227 (PMC6787489; doi:10.3389/fnbeh.2019.00227)
Supplement: Supplementary file 1 [file Table_1.DOCX]

**
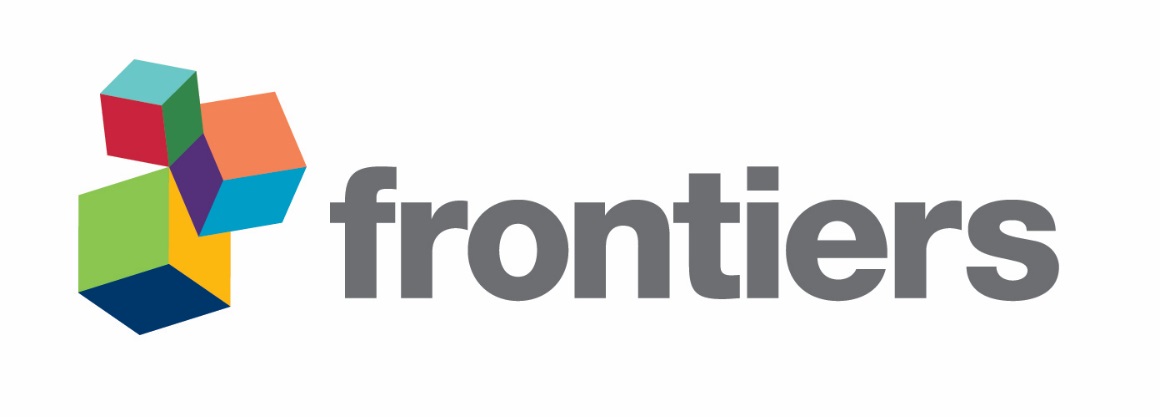
**

**SUPPLEMENTARY FIGURES**

**Supplementary Figure 1. NAc core Subject #2. A)** This probe was in medial rostral NAc core (+2.4 mm from bregma). **B)** MATLAB annotated signal and peak analysis for the first mating bout. Blue rectangles indicate peaks determined by a threshold value of half of the root-mean-square of the normalized signal**.** Purple circles (startMountI) indicate the start of a mount that results in an intromission. Aqua circles (startIntromission) indicate the start of a penile intromission from a male. Green circles (endMountI) indicate the end of a mount that resulted in an intromission. Red circles (endIntromission) indicate the end of an intromission. **C)** Mounts with subsequent intromissions were collapsed across the first 5 min of the sex test. These mounts with intromission had a coincident increase in glutamate (blue signal) that peaked just after the start of the mount. There were no mounts without subsequent intromission in the first 5 min.

**Supplementary Figure 2. NAc core Subject #3. A)** This probe was in the medial NAc core (+1.8 mm from bregma). **B)** MATLAB annotated signal and peak analysis for the first mating bout. Blue rectangles indicate peaks determined by a threshold value of half of the root-mean-square of the normalized signal**.** Purple circles (startMount) indicate the start of a mount that does not result in an intromission. Aqua circles (startMountI) indicate the start of a mount that results in a subsequent intromission from a male. Pink circles (startIntromission) indicate the start of a penile intromission from the male. Green circles (endMountI) indicate the end of a mount that resulted in an intromission. Red circles (endIntromission) indicate the end of an intromission. **C)** Mounts with subsequent intromissions were collapsed across the first 5 min of the sex test. These mounts with intromission had a coincident increase in glutamate (blue signal) that peaked 3 sec after the start of the mount. No coincident signal was seen in glutamate among mounts without intromission (red signal).

**Supplementary Figure 3. NAc core Subject #4. A)** This probe was in the medial NAc core (+1.5 mm from Bregma). **B)** MATLAB annotated signal and peak analysis for the first mating bout. Blue rectangles indicate peaks determined by a threshold value of half of the root-mean-square of the normalized signal**.** Purple circles (startMount) indicate the start of a mount that does not result in an intromission. Aqua circles (startMountI) indicate the start of a mount that results in a subsequent intromission from a male. Pink circles (startIntromission) indicate the start of a penile intromission from the male. Green circles (endMountI) indicate the end of a mount that resulted in an intromission. Red circles (endIntromission) indicate the end of an intromission. **C)** Mounts with subsequent intromissions were collapsed across the first 5 min of the sex test. These mounts with intromission had a coincident increase in glutamate (blue signal) that peaked about 1 sec after the start of the mount. No peak signal was seen in glutamate during the single mount without intromission (red signal) recorded during the first 5 min.

**Supplementary Figure 4. NAc shell Subject #2. A)** This probe was in the rostral NAc ventral shell (+2.6 mm from Bregma). **B)** MATLAB annotated signal and peak analysis for the first mating bout. Blue rectangles indicate peaks determined by a threshold value of half of the root-mean-square of the normalized signal. Blue circles (startMountI) indicate the start of a mount that results in an intromission. Magenta circles (startIntromission) indicate the start of a penile intromission from a male. Green circles (endMountI) indicate the end of a mount that resulted in an intromission. **C)** Although there are significantly more glutamate peaks associated with mounts that resulted in intromission, there was no coincident timing of those peaks (blue signal), a finding similar to that of mounts without intromission (red signal).

**Supplementary Figure 5. NAc shell Subject #3. A)** This probe was in the NAc ventral shell (+2.1 mm from Bregma). **B)** MATLAB annotated signal and peak analysis for the first mating bout. Blue rectangles indicate peaks determined by a threshold value of half of the root-mean-square of the normalized signal. Blue circles (startMountI) indicate the start of a mount that results in an intromission. Magenta circles (startIntromission) indicate the start of a penile intromission from a male. Green circles (endMountI) indicate the end of a mount that resulted in an intromission. **C)** Although there are significantly more glutamate peaks associated with mounts that resulted in intromission, there was no coincident timing of those peaks (blue signal), a finding similar to that of mounts without intromission (red signal).

**Supplementary Figure 6. NAc shell Subject #4. A)** This probe was in the medial NAc shell (+1.8 mm from Bregma). **B)** MATLAB annotated signal and peak analysis for the first mating bout. Blue rectangles indicate peaks determined by a threshold value of half of the root-mean-square of the normalized signal. Blue rectangles indicate peaks determined by a threshold value of half of the root-mean-square of the normalized signal. Red circles (EndAI) indicate the end of an anogenital investigation from the male. Yellow circles (endIntromission) indicate the end of a penile intromission from a male. Green circles (endMountI) indicate the end of a mount that resulted in an intromission. Aqua circles (startAI) indicate the start of anogenital investigation from the male. Blue circles (startIntromission) indicate the start of a penile intromission from a male. Magenta circles (startMountI) indicate the start of a mount that results in an intromission. **C)** Although there are significantly more glutamate peaks associated with mounts that resulted in intromission, there was no coincident timing of those peaks (blue signal), a finding similar to that of mounts without intromission (red signal).
